# Programmed cell death markers in COVID-19 survivors with and without sepsis

**DOI:** 10.3389/fimmu.2025.1535938

**Published:** 2025-02-20

**Authors:** Chandra Shekar Mallarpu, Srinivasa Ikswaja Chelluri, Tapaswi Krishna Katragadda, Maneendra Singarapu, Lakshmi Kiran Chelluri, Charitha Madiraju

**Affiliations:** ^1^ Department of Transplant Immunology and Stem Cell Lab, Global Medical Education and Research Foundation, Hyderabad, India; ^2^ Bharati Vidyapeeth Medical College, Deemed to be University, Pune, Maharashtra, India; ^3^ Department of Respiratory and Critical Care Medicine, Gleneagles Hospitals, Hyderabad, India; ^4^ Department of Medical Sciences, Hackensack Meridian School of Medicine, Nutley, NJ, United States; ^5^ Department of Pharmaceutical Sciences, Marshall B. Ketchum University College of Pharmacy, Fullerton, CA, United States

**Keywords:** sepsis, COVID-19, programmed cell death, caspase-3, caspase-1, MLKL, p62/SQSTM1, cytokines

## Abstract

**Introduction:**

Sepsis remains a leading cause of mortality, especially in COVID-19 patients, due to delayed diagnosis and limited therapeutic options. While the mechanisms of programmed cell death (PCD) in COVID-19 and sepsis are complex, understanding the molecular markers involved in these processes may aid in assessing disease severity. This study aimed to investigate the roles of PCD markers, inflammatory cytokines, and MHC molecules in distinguishing disease severity in COVID-19 patients with and without sepsis.

**Methods:**

The study involved adult patients (≥18 years) who survived COVID-19, grouped into four cohorts: COVID-19 with sepsis (C19wSepsis), COVID-19 without sepsis (C19NoSepsis), sepsis alone, and healthy controls. Serum and peripheral blood mononuclear cells (PBMCs) from each cohort were analyzed using enzyme-linked immunosorbent assay (ELISA) and flow cytometry. PCD markers (caspase-3, caspase-1, MLKL, LC3B, p62/SQSTM1), inflammatory cytokines (IL-1-beta, IFN-gamma), and MHC molecules (MHC I-A, MHC II-DRB1) were assessed. Statistical analyses were performed to evaluate differences in marker levels between and within cohorts.

**Results:**

The analysis identified two distinct molecular signatures associated with disease severity. The first signature, characterized by elevated levels of secreted markers of PCD, IL-1-beta, IFN-gamma, MHC I-A and MHC II-DRB1, was common to the C19wSepsis and C19NoSepsis cohorts. The second signature, which was more prominent in the cellular markers of PCD (caspase-1, caspase-3, MLKL, p62/SQSTM1), was uniquely associated with the C19wSepsis cohort.

**Conclusion:**

These findings provide insight into the molecular signatures distinguishing immune responses in COVID-19-related sepsis and may serve as valuable biomarkers for assessing disease severity, while guiding therapeutic interventions in critical care settings.

## 1 Introduction

Sepsis is a critical illness in response to an infection that can progress to multiple organ dysfunction syndrome (MODS) (1–4). The hyperinflammatory phase observed in the initial stages of sepsis leads to an immunosuppressive state due to dysfunctional immune response mechanisms (5–7). Intricate mechanisms of immune response play a key role in life-threatening infections, including the COVID-19 pandemic-causing virus, SARS-CoV-2 (8–13). Early diagnosis and urgent therapeutic intervention are crucial for sepsis and COVID-19 patient survival; clinical heterogeneity, especially in patients with underlying comorbidities, influences infectious disease severity and risk of MODS (14–18). The timely discovery of COVID-19 vaccines is a breakthrough savior during the recent COVID-19 pandemic period (19–21). Programmed Cell death (PCD) plays a significant role in sepsis (22–30). PCD mechanisms of apoptosis, pyroptosis, necroptosis and autophagy have been implicated in the pathogenesis of COVID-19 infections, sepsis, sepsis-induced organ dysfunction, disease severity and cytokine-induced responses (31–37). COVID-19 infection, unlike influenza infection, was reported as not characterized by a cytokine storm, instead of markedly impaired immune effector cell function indicative of profound immunosuppression (38, 39). Despite advancements in sepsis research, there is still a lack of understanding of the immunopathogenesis and immunological drug targets of sepsis, further exploration of which paves the way for innovative therapeutic interventions (40). To this end, the establishment of PCD marker profile is one approach that might identify potential biomarkers of PCD in COVID-19 patients with and without sepsis and help unravel the differential clinical outcomes in different subsets of sepsis.

The goal of our study was to identify PCD markers [caspase-3 for apoptosis, caspase-1 for pyroptosis, mixed-linked lineage kinase-like domain (MLKL) for necroptosis, and LC3B and p62/SQSTM1 for autophagy], cytokines [interleukin-1-beta (IL-1-beta) and interferon-gamma (IFN-gamma)], and major histocompatibility molecules (MHCs: MHC I-A, MHC II-DRB1), in samples derived from patients with COVID-19 with or without sepsis, sepsis alone, and normal healthy subjects that served as controls. Secreted and intracellular levels of PCD markers were investigated by Enzyme-linked immunosorbent assay (ELISA) and flow cytometry analysis of serum and peripheral blood mononuclear cells (PBMCs), respectively, derived from COVID-19 with sepsis patients, COVID-19 with no sepsis patients, and patients with sepsis alone versus controls. The significance of this initial research study was to define the potential of PCD markers in determining the clinical outcomes of COVID-19 with and without sepsis.

## 2 Materials and methods

### 2.1 Materials

The materials used in this study were as follows: Triton™ X-100 (catalog no. P212121, Sigma Aldrich, Japan), phosphate-buffered saline (PBS, catalog no. F180725DS; Euroimmun-USA), acetone (catalog no. SF6F660388; Merck Life Science), para-formaldehyde (catalog no. GRM3660; Himedia-India), bovine serum albumin (BSA, catalog no. A2153-504 Sigma-USA), trypan blue (0.1%, catalog no. T8154# RNBD 8640, Sigma-Aldrich), Ficoll Histopaque (catalog no. 10771, MP Biomedicals, LLC, France), and propidium iodide (catalog no. 006990-42, e-biosciences). ELISA kits for the markers included in this study were purchased from Wuhan Fine Biotech, China, and included the following: human caspase-1 (catalog no. EH0595), caspase-3 (catalog no. EH0546), MLKL (catalog no. OKEH03401), p62/SQSTM1 (catalog no. EH10842), MHC I-A (catalog no. RK01844 ABclonal), MHC II-DRB1 (catalog no. RK01847 ABclonal), IL-1-beta (catalog no. EH0185) and IFN-gamma (catalog no. EH0164). The primary antibodies included apoptosis/necroptosis antibodies (sampler kit # 92570T Cell Signaling Technology, USA), rabbit monoclonal antibody against caspase-1 (CST, clone D7F10, catalog no. #2225S), rabbit monoclonal antibody against caspase-3 (CST, clone D3R6Y, catalog no. #14220), rabbit monoclonal antibody against MLKL (CST, clone D216N, catalog no. #14993), rabbit polyclonal antibody against p62/SQSTM1 (Fine Test, clone 7074P2, catalog no. #FNab06086), and rabbit monoclonal antibody against LC3B (CST, clone D3U4C catalog no. 12741S). The secondary antibodies used were anti-rabbit FITC (catalog no. 349031) and Alexa Fluor 546 (catalog no. A-21085, Invitrogen).

### 2.2 Study design, inclusion & exclusion criteria

This study was approved by the Institutional Ethics Committee (IEC ref. # GGH/BS/2018-01) of Gleneagles Global Hospitals, Hyderabad TS, India, and the Institutional Review Board (IRB ref. #OHRP IORG NO 000 1030, IRB NO 0000 1418) of Marshall B. Ketchum University, Fullerton, CA, USA. This study was conducted in accordance with the principles of the Declaration of Helsinki Declaration. Adult patients (age ≥18 years) that survived COVID-19 infection and/or bacterial sepsis were included in the study. COVID-19-positive patients with sepsis (C19wSepsis), COVID-19-positive patients without sepsis (C19NoSepsis or C19 alone), Sepsis Alone (Sepsis alone), and normal healthy subjects (controls) were included in the study. Written informed consent was obtained from all the subjects enrolled in the study.

**Figure 1:**
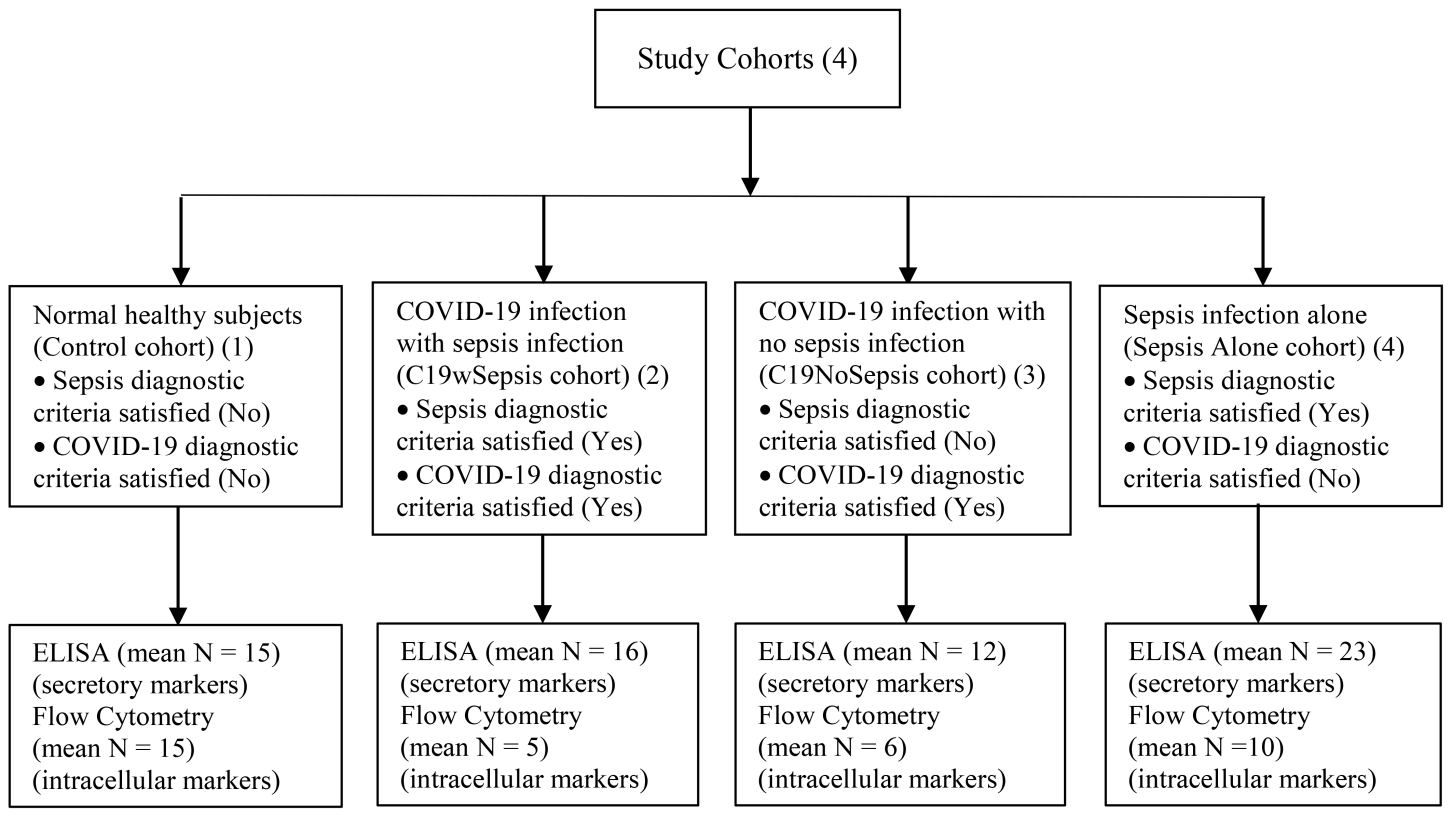
Flowchart illustrating the categorization of subjects into the four clinical cohorts and methodological approaches involving the four cohorts.

Patients with sepsis were categorized according to the Third International Consensus Definition for sepsis (16). Patients were classified as having sepsis if they satisfied the following two or more diagnostic criteria for sepsis: (1) body temperature > 38°C or < 36°C (2) heart rate > 90 beats per minute; (3) respiratory rate >20 breaths per minute; and (4) white blood cell (WBC) count >12,000/cu mm, < 4,000/cu mm (16). COVID-19 infection is confirmed to be positive upon testing for SARS-CoV-2, the causative pathogen of COVID-19, using an RT-PCR-based diagnostic method (41). Patients on organ transplantation, those with an onset of sepsis syndrome for more than 24 -48 h, other infections, those undergoing anti-retroviral therapy, malignant diseases, aseptic inflammation and miscellaneous conditions related to post-transplant were excluded from the study. Patients who fulfilled the sepsis diagnostic criteria were on antibiotic treatment [(Ceftriaxone, Monocef^®^ 1000 mg/vial injection (one or two doses) alone or in combination with flucloxacillin sodium, A-Flox^®^ 500 mg/vial injection (one or two doses) depending on the severity of the confirmed infection]. Subjects were recruited into the study and were categorized into the following four clinical cohorts: (1) patients diagnosed with COVID-19 infection along with sepsis [COVID-19 with sepsis, denoted as C19wSepsis]; (2) patients diagnosed with COVID-19 infection alone with no sepsis [COVID-19 with no sepsis, denoted as C19NoSepsis or C19 alone]; (3) patients diagnosed with sepsis alone [denoted as Sepsis Alone in Figures and Tables]; and (4) normal healthy subjects with no underlying disease [controls]. The four study cohorts along with parameters for diagnosis of sepsis along with inclusion and exclusion criteria are listed in **Table 1**. **Figure 1** illustrates the categorization of subjects into the four clinical cohorts and methodological approaches involving the four cohorts.

**Table 1:** The four clinical cohorts included in the study, the diagnostic criteria and exclusion criteria are listed below.

Clinical condition	Mean values								
	Age (yr)	qSOFA	APACHE II	Temp (°C)	Respiratory rate (breaths/ min)	Heart rate (beats/ min)	WBC Count (cells/ cu mm)	ICU Admission	Duration of Symptoms (days)
								Treatment Regimen	
Normal healthy subjects (Control cohort)	28	NA	NA	>38	18	80	5211	NA	NA
COVID-19 with sepsis infection (C19wSepsis)	40	2	>15	37.2	22.8	113	14339	Antiviral	4
COVID-19 with no sepsis infection (C19NoSepsis)	37	1	>15	34.0	20.9	103	7740	Antiviral	3
Sepsis infection alone (Sepsis Alone)	34	1	>15	36.7	24.2	117	13765	Antimicrobial	3

Mean values are represented for each parameter indicated as part of diagnostic criteria. Normal healthy subjects with no underlying disease [Control cohort]. Patients diagnosed with COVID-19 infection along with sepsis infection [COVID-19 with sepsis, C19wSepsis cohort]. Patients diagnosed with COVID-19 infection with no sepsis infection [COVID-19 with no sepsis, C19NoSepsis cohort]. Patients diagnosed with sepsis infection alone [Sepsis Alone cohort].

Inclusion Criteria: Sepsis patients confirmed with an infection had met with two or more of the inclusion criteria which include, (1) body temperature > 38°C or < 36°C (2) heart rate > 90 beats per minute (3) respiratory rate > 20 breaths per minute (4) white blood cell (WBC) count > 12,000/cu mm, < 4,000/cu mm. NA, not applicable.

Exclusion Criteria: Other infections, malignant diseases, aseptic inflammation, and miscellaneous conditions related to post-transplant were excluded.

### 2.3 Experimental methodology and data analysis

#### 2.3.1 Sample collection and isolation of peripheral blood mononuclear cells (PBMCs)

The methodology for sample collection and isolation of PBMCs was performed as described previously (23). Peripheral blood from vein-puncture (5.0 mL) was collected separately in EDTA and plain vacutainers post-antibiotic dose (18-24 h after antibiotic administration). The samples were collected during the pandemic from 2019 to 2022. Serum and peripheral blood mononuclear cells (PBMCs) were separated immediately by centrifugation, transferred into individual cryotubes, and stored at -80°C until further processing. Density gradient centrifugation using Ficoll Histopaque (MP Biomedicals, LLC, France #190837) was used to isolate PBMCs from whole blood (23). Briefly, whole blood samples collected from the four study cohorts were carefully layered on top of the Ficoll solution (3:1 ratio) and centrifuged at room temperature (RT, 15-25°C) for 30 min at 201 x g. PBMCs found at the interface between plasma and Ficoll were collected, washed, and resuspended in an appropriate volume of PBS.

#### 2.3.2 Enzyme-linked immunosorbent assay (ELISA)

Serum samples derived from the four study cohorts [C19wSepsis, C19NoSepsis (C19 alone), sepsis alone, and healthy controls] were analyzed by ELISA using commercially available kits for the detection of caspase-1, caspase-3, MLKL, p62/SQSTM1, IL-1-beta, IFN-gamma, MHC I-A, and MHC II-DRB1 as described previously (23). The sample size for each cohort is provided in **Table 2** as part of the ELISA data. ELISA experiments for the above markers were performed in 96-well plates, according to the manufacturer’s instructions. Secreted levels of each of the above markers were quantified in samples derived from healthy controls, C19 patients with or without sepsis, and sepsis alone.

**Table 2:** Normalized ELISA data showing mean fold-change in serum markers of cell death, cytokines and MHCs with respect to controls observed in the four study cohorts.

Cohort No.	Clinical cohort	Mean±SEM (N)								
		Mean Sample Size	Caspase-1	Caspase-3	MLKL	p62/ SQSTM1	IL-1-beta	IFN- gamma	MHC I-A	MHC II- DRB1
1	Normal healthy subjects (Control)	N=15	1 ± 0.15 (N=18)	1 ± 0.16 (N=15)	1 ± 0.105 (N=21)	1 ± 0.14 (N=18)	1 ± 0.21 (N=14)	1 ± 0.16 (N=10)	1 ± 0.93 (N=10)	1 ± 0.196 (N=14)
2	COVID-19 infection with Sepsis infection (C19wSepsis)	N=16	0.62 ± 0.08 (N=14)	2.82 ± 0.38 (N=17)	1.19 ± 0.25 (N=15)	0.91 ± 0.11 (N=17)	5.45 ± 0.63 (N=16)	3.15 ± 0.395 (N=17)	14.2 ± 1.58 (N=17)	8.11 ± 0.32 (N=17)
3	COVID-19 infection with no Sepsis infection (C19NoSepsis)	N=12	0.296 ± 0.06 (N=10)	3.10 ± 0.19 (N=12)	1.46 ± 0.14 (N=12)	0.23 ± 0.01 (N=7)	2.47 ± 0.41 (N=14)	0.93 ± 0.24 (N=11)	1.9 ± 0.66 (N=14)	3.38 ± 0.35 (N=17)
4	Sepsis infection alone (Sepsis Alone)	N=23	0.63 ± 0.07 (N=23)	4.92 ± 0.84 (N=24)	4.38 ± 1.0 (N=24)	0.58 ± 0.05 (N=21)	1.595 ± 0.11 (N=23)	2.11 ± 0.13 (N=24)	7.12 ± 0.81 (N=24)	6.19 ± 0.03 (N=24)

PCD markers in serum samples derived from patients (denoted as C19wSepsis, C19NoSepsis, and Sepsis Alone) and healthy controls were measured using ELISA. Secreted levels of caspase-3 (marker for apoptosis), caspase-1 (marker for pyroptosis), MLKL (marker for necroptosis), p62/SQSTM1 (marker for autophagy), IL-1-beta (proinflammatory cytokine), IFN-gamma (antiviral cytokine), antigen processing and presentation molecules, MHC I-A and MHC II-DRB1 were detected in serum samples using commercially available ELISA kits. The concentration of each secreted marker was interpolated from the measured values using the respective standard graphs for each marker. Data were corrected for dilution factors. Data and statistical analyses were performed to compare the secreted levels of immunological markers between clinical cohorts and between immunological markers within a given clinical cohort. First, ELISA data will be compared between clinical cohorts (C19wSepsis, C19NoSepsis, Sepsis Alone and healthy controls) for each of the markers mentioned above, namely, caspase-1, caspase-3, MLKL, p62/SQSTM1, IL-1-beta, IFN-gamma, MHC I-A, and MHC II-DRB1. Second, ELISA data within each clinical cohort are presented to compare the above-mentioned markers.

#### 2.3.3 Flow cytometry

PBMCs isolated from the four study cohorts [C19wSepsis, C19NoSepsis (C19 alone), Sepsis Alone, and healthy controls] were prepared and analyzed by flow cytometry (23). The sample size for each cohort is provided in **Table 3** as part of the flow cytometry data. Briefly, the cells were permeabilized with organic solvents (methanol and acetone), followed by the addition of 5% Triton™ X-100 detergent (Sigma Aldrich, Japan) and 1 mL of 4% paraformaldehyde (Hi-Media-India) to the prepared PBMCs. The cells were then incubated for 10 min at RT. Samples were washed with 1X PBS (Sigma-Aldrich), followed by the addition of 1 mL of 0.1% Triton™ X-100 and incubation of the samples for an additional 30 min. Cells were blocked using 1% BSA for 30 min, and cell count and viability were determined using the Trypan Blue exclusion method (Sigma-Aldrich). Approximately, 1x10^6^ cells were stained with the following primary antibodies: caspase-1, caspase-3, MLKL, p62/SQSTM1 and LC3B (Cell Signaling Technology, USA), and cells were incubated overnight at 4°C; anti-rabbit- and FITC-conjugated secondary antibodies, and PI (1:100 dilution) were added to the samples and incubated for an additional 45 min at RT. Cells were acquired using a BD FACSCalibur™ flow cytometer [Beckman, Dickinson BD FACSCalibur E6210; Beckman Dickinson] using the BD CellQuest software.

**Table 3:** Normalized flow cytometry data showing mean fold-change in percentage shift of cell death markers with respect to controls observed in the four study cohorts.

Cohort No.	Clinical cohort	Mean±SEM (N)					
		Mean Sample Size	Caspase-1	Caspase-3	MLKL	LC3B	p62/ SQSTM1
1	Normal healthy subjects (Control)	(N=15)	1 ± 0.17 (N=14)	0.81 ± 0.176 (N=16)	1 ± 0.15 (N=14)	1 ± 0.12 (N=15)	1 ± 0.17 (N=18)
2	COVID-19 infection with Sepsis infection (C19wSepsis)	(N=5)	4.92 ± 0.96 (N=4)	6.33 ± 0.77 (N=5)	9.34 ± 0.27 (N=4)	7.83 ± 0.24 (N=5)	14.8 ± 0.63 (N=5)
3	COVID-19 infection with no Sepsis infection (C19NoSepsis)	(N=6)	2.45 ± 0.37 (N=5)	0.28 ± 0.04 (N=10)	1.47 ± 0.8 (N=5)	0.26 ± 0 (N=5)	0.65 ± 0.11 (N=6)
4	Sepsis infection alone (Sepsis Alone)	(N=10)	4.96 ± 0.63 (N=12)	0.16 ± 0 (N=10)	1.42 ± 0.31 (N=11)	2.09 ± 0.46 (N=11)	3.9 ± 0.52 (N=8)

FACS analysis was performed using FITC-conjugated antibodies against caspase-3 (for apoptosis), caspase-1 (for pyroptosis), MLKL (for necroptosis), LC3B and p62 (for autophagy). Like the ELISA data, flow cytometry data will first be compared between clinical cohorts for each of the cell death markers mentioned above, followed by flow cytometry data presentation to compare the cell death markers within each clinical cohort for the above markers. Graphical and statistical analyses of normalized percentage (%) of gated cell population data were performed on data derived from flow cytometry analyses of PBMC samples from patient cohorts (C19wSepsis, C19NoSepsis, and Sepsis Alone) and healthy subjects (controls) for each cell death marker.

#### 2.3.4 Data and statistical analyses

Data and statistical analyses were performed using GraphPad Prism, version 7.03. Absorbance (OD 450 nm) values for ELISA experiments for patient and control samples were interpolated by regression analysis of the respective standards for PCD and immune regulatory markers, including caspase-1, caspase-3, MLKL, p62/SQSTM1, IL-1-beta, IFN-gamma, MHC I-A, and MHC II-DRB1. Flow cytometry analyses of % gated cell population data, represented as mean fold-change with respect to controls, for cell death markers observed for different clinical conditions of patient cohorts and healthy controls. Representative histogram plots of cell death markers derived from flow cytometry analysis of PBMCs derived from the Control, Sepsis Alone, C19NoSepsis and C19wSepsis cohorts. The interpolated values were corrected for dilution factors. Percentage shifts in mean fluorescence intensity values were used for data derived from the flow cytometry analyses of PCD markers (caspase-1, caspase-3, MLKL, p62, and LC3B). All the data were normalized to the respective control samples. Non-parametric Kruskal-Wallis one-way analysis of variance (ANOVA) combined with multiple comparisons or non-parametric Mann-Whitney test analyses were used as appropriate for statistical analyses of data derived from ELISA and flow cytometry experiments. Statistical significance was set at *p*-values < 0.05.

## 3 Results

### 3.1 Secreted cell death markers, cytokines and MHCs between clinical cohorts

**Figure 2:**
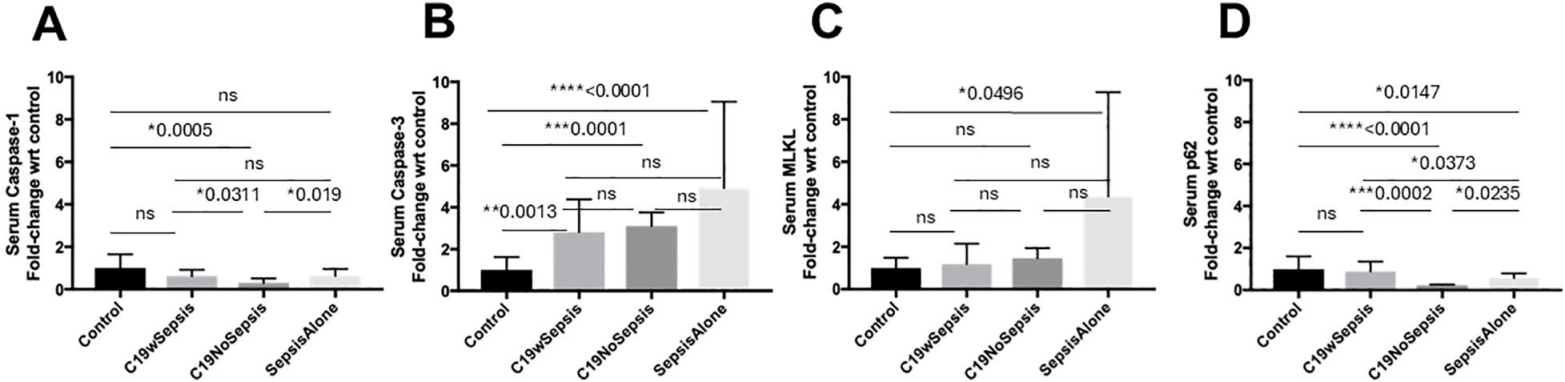
ELISA of secreted cell death markers between clinical cohorts. Normalized data from ELISA of serum samples from Control, C19wSepsis, C19NoSepsis and Sepsis Alone cohorts. Clinical cohorts (X-axis) versus fold-change in serum levels of cell death markers with respect to controls (Y-axis) are represented for caspase-1 **(A)**, caspase-3 **(B)**, MLKL **(C)** and p62 **(D)**. All data expressed as mean±SEM and statistical significance shown for data where *p* < 0.05. Note: Statistical significance level is indicated by the number of asterisks. * *p* ≤ 0.05; ** *p* ≤ 0.01; *** *p* ≤ 0.001; **** *p* ≤ 0.0001; ns, non-significant, *p* > 0.05.

**Figure 3:**
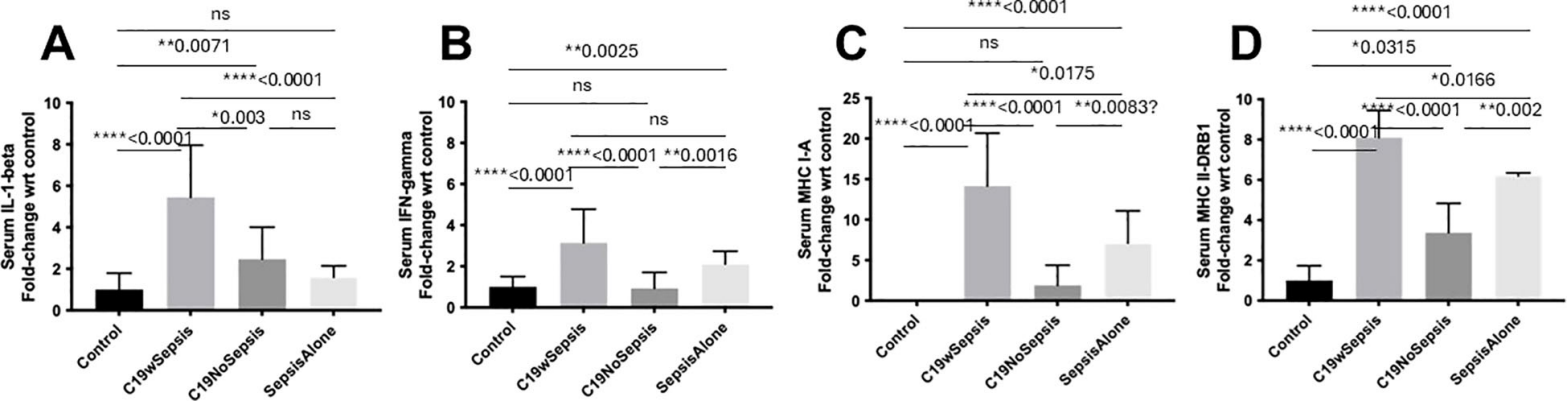
ELISA of secreted cytokines and MHCs between clinical cohorts. Normalized data from ELISA of serum samples from Control, C19wSepsis, C19NoSepsis and Sepsis Alone cohorts. Clinical cohorts (X-axis) versus fold-change in serum levels of cytokines and MHCs with respect to controls (Y-axis) are represented for IL-1-beta **(A)**, IFN-gamma **(B)**, MHC I-A **(C)** and MHC II-DRB1 **(D)**. All data expressed as mean±SEM and statistical significance shown for data where *p* < 0.05. Note: Statistical significance level is indicated by the number of asterisks. * *p* ≤ 0.05; ** *p* ≤ 0.01; **** *p* ≤ 0.0001; ns, non-significant, p > 0.05.

The results of an extensive ELISA analysis of the levels of the eight different markers including caspase-1, caspase 3, MLKL, p62/SQSTM1, IL-1-beta, IFN-gamma, MHC I-A and MHC II-DRB1 revealed the difference among cohorts and controls (healthy subjects) in molecular signatures (**Figures 2**, **3**; **Table 2**).

The secreted caspase-1 varied among different cohorts and controls in a distinct pattern (**Figure 2A**; **Table 2**). No significant difference observed in the mean fold-change in secreted caspase-1 levels between the control, C19wSepsis, and Sepsis Alone cohorts (*p*>0.05). However, the C19NoSepsis cohort showed a significant 3-fold decrease in secreted caspase-1 levels compared to the control group (**p=0.0025). A 2-fold significant increase in secreted caspase-1 in C19wSepsis cohort was observed compared to C19NoSepsis cohort (*p=0.0311), and the Sepsis Alone cohort had a 2-fold significant increase compared to C19NoSepsis cohort (*p=0.019). There was no significant difference in the secreted caspase-1 between the C19wSepsis and Sepsis Alone cohorts (*p*>0.05).

The secreted levels of caspase-3 in different cohorts varied with controls (**Figure 2B**; **Table 2**). The mean fold-change in the secreted levels of caspase-3 showed a significant increase in three clinical cohorts compared to the control cohort: C19wSepsis (> 2-fold increase, **p=0.0013), C19NoSepsis (> 3-fold increase, ***p=0.0001), and Sepsis Alone (> 4-fold increase, ****p<0.0001). However, no significant differences were observed between the C19wSepsis vs. C19NoSepsis vs. Sepsis Alone cohorts in the secreted caspase-3 levels (*p*>0.05).

The secreted levels of MLKL did not show noticeable differences among COVID-19 cohorts and controls (**Figure 2C**; **Table 2**). There were no significant differences observed in the mean fold-change values of secreted MLKL levels between the control group and the C19wSepsis or C19NoSepsis cohorts (*p*>0.05). Comparison of the control versus Sepsis Alone cohorts showed an increase in the secreted MLKL levels in the Sepsis Alone cohort compared to the control group (> 4-fold increase in Sepsis Alone, *p=0.0496). There were no significant differences in the secreted MLKL among the three cohorts of C19wSepsis, C19NoSepsis, and Sepsis Alone (*p*>0.05).

The secreted p62 levels varied markedly among different cohorts and controls (**Figure 2D**; **Table 2**). While no significant difference in the mean-fold change in secreted p62 levels were observed between the control group and the C19wSepsis cohort (p>0.05), a significant decrease in secreted p62 levels in the C19NoSepsis (> 4-fold decrease, ****p<0.0001) and Sepsis Alone cohorts (> 1.5-fold decrease, *p=0.0147) were observed compared to controls. Furthermore, comparison between the clinical cohorts showed a significant decrease in secreted p62 in C19NoSepsis (> 4-fold decrease, ***p=0.0002) and Sepsis Alone (> 2-fold decrease, *p=0.0373) cohorts compared to C19wSepsis, while secreted p62 levels showed a significant decrease in Sepsis Alone cohort compared to that in C19NoSepsis cohort (> 2-fold decrease, *p=0.0235).

The secreted IL-1-beta levels followed a noticeable pattern among different cohorts and controls (**Figure 3A**; **Table 2**). The mean fold-change in the secreted levels of IL-1-beta were significantly higher in the C19wSepsis cohort (> 5-fold increase, ****p<0.0001) and C19NoSepsis cohort (> 2-fold increase, **p=0.0071) but not in Sepsis Alone cohort (> 1.5-fold increase, *p*>0.05) compared to controls. Comparison of IL-1-beta between the cohorts showed a significant increase in the secreted IL-1-beta levels in the C19wSepsis cohort compared to the C19NoSepsis (> 2-fold increase, **p=0.003) and Sepsis Alone (> 3-fold increase, ****p<0.0001) cohorts. No significant difference in the secreted IL-1-beta observed between the C19NoSepsis and Sepsis Alone cohorts (> 1.5-fold increase, *p*>0.05) cohorts.

The secreted levels of IFN-gamma also followed a pattern among different cohorts and controls (**Figure 3B**; **Table 2**). While the mean fold-change in secreted IFN-gamma levels showed a significant increase in IFN-gamma in the C19wSepsis (> 3-fold increase, ****p<0.0001) and Sepsis Alone (> 2-fold increase, **p=0.0025) cohorts compared to the control group, IFN-gamma levels in the C19NoSepsis cohort were not significantly different from that in the control cohort (*p*>0.05). A significant increase in the secreted IFN-gamma was observed in C19wSepsis compared to C19NoSepsis (> 3-fold increase, ****p<0.0001) and Sepsis Alone (about 1.5-fold increase, *p*>0.05) cohorts. Furthermore, secreted IFN-gamma was significantly higher in Sepsis Alone cohort compared to C19NoSepsis (> 2-fold increase, **p=0.0016) cohort.

The secreted MHC I-A varied markedly among different cohorts and controls (**Figure 3C**; **Table 2**). The mean fold-change in MHC I-A levels were significantly higher in the C19wSepsis cohort (> 14-fold increase, ****p<0.0001) and Sepsis Alone group (> 7-fold increase, ****p<0.0001) compared with the control group. There was no significant increase in MHC I-A levels between the C19NoSepsis and control cohorts (*p*>0.05). Comparison of secreted MHC I-A between clinical cohorts also showed a significant increase in MHC I-A in the C19wSepsis cohort compared to C19NoSepsis (> 7-fold increase, ****p<0.0001) and Sepsis Alone (about a 2-fold increase, *p<0.0175) cohorts. The Sepsis Alone cohort showed a significant increase in secreted MHC I-A versus the C19NoSepsis cohort (> 3-fold increase, **p=0.0083).

The secreted MHC II-DRB1 showed a distinct pattern among different cohorts and controls (**Figure 3D**; **Table 2**). A significant increase in the mean fold-change in MHC II-DRB1 levels in the C19wSepsis cohort (> 8-fold increase, ****p<0.0001), C19NoSepsis cohort (> 3-fold increase, *p=0.0315), and Sepsis Alone group (> 6-fold increase, ****p<0.0001) were observed compared to the control group. Comparison of MHC II-DRB1 levels between cohorts also showed a significant increase in secreted MHC II-DRB1 in the C19wSepsis versus C19NoSepsis (> 2-fold increase, ****p<0.0001) and Sepsis Alone (*p=0.0166) cohorts, while the Sepsis Alone cohort showed a significant increase in secreted MHC II-DRB1 compared to C19NoSepsis cohort (> 1.5-fold increase, **p=0.002).

### 3.2 Secreted cell death markers, cytokines and MHCs within each clinical cohort

**Figure 4:**
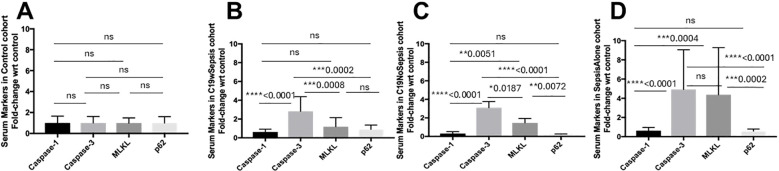
ELISA of secreted cell death markers within each clinical cohort. Normalized data from ELISA of serum samples from Control, C19wSepsis, C19NoSepsis and Sepsis Alone cohorts. Cell death markers (X-axis) versus fold-change in serum levels of cell death markers with respect to controls (Y-axis) are represented for Control **(A)**, C19wSepsis **(B)**, C19NoSepsis **(C)** and Sepsis Alone **(D)** cohorts. All data expressed as mean±SEM and statistical significance shown for data where *p* < 0.05. Note: Statistical significance level is indicated by the number of asterisks. * *p* ≤ 0.05; ** *p* ≤ 0.01; *** *p* ≤ 0.001; **** *p* ≤ 0.0001; ns, non-significant, *p* > 0.05.

**Figure 5:**
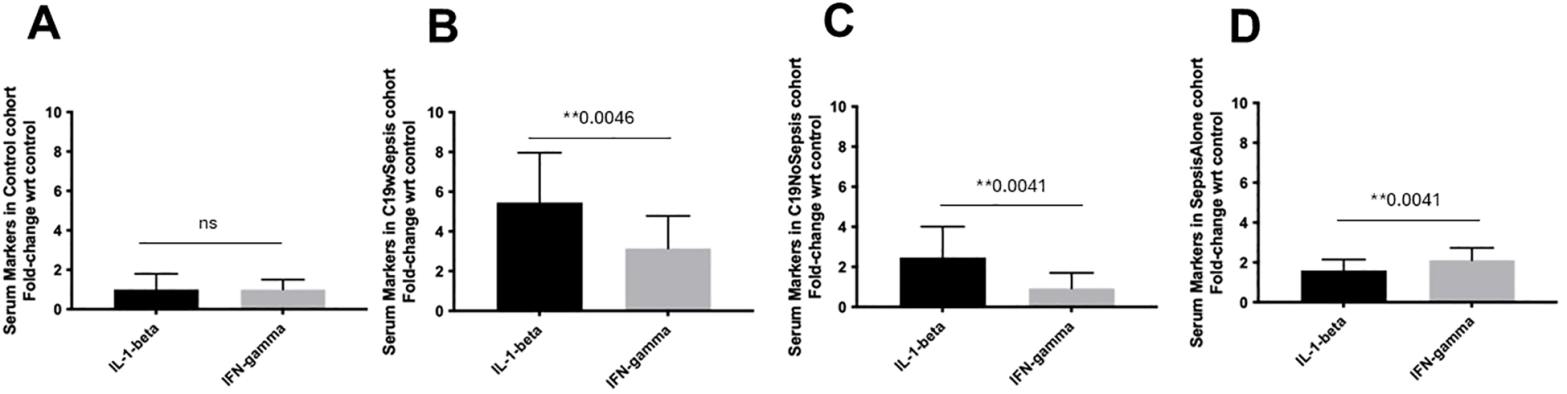
ELISA of secreted cytokine markers within each clinical cohort. Normalized data from ELISA of serum samples from Control, C19wSepsis, C19NoSepsis and Sepsis Alone cohorts. Cytokine markers (X-axis) versus fold-change in serum levels of cytokine markers with respect to controls (Y-axis) are represented for Control **(A)**, C19wSepsis **(B)**, C19NoSepsis **(C)** and Sepsis Alone **(D)** cohorts. All data expressed as mean±SEM and statistical significance shown for data where *p* < 0.05. Note: Statistical significance level is indicated by the number of asterisks. ** *p* ≤ 0.01; ns, non-significant, *p* > 0.05.

**Figure 6:**
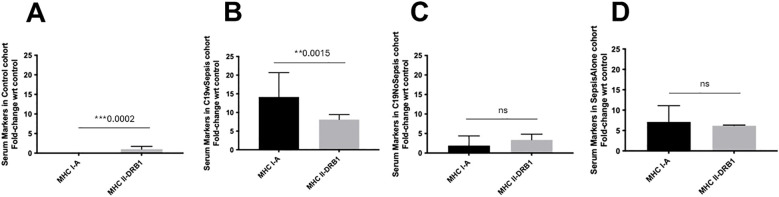
ELISA of secreted MHC markers within each clinical cohort. Normalized data from ELISA of serum samples from Control, C19wSepsis, C19NoSepsis and Sepsis Alone cohorts. MHC markers (X-axis) versus fold-change in serum levels of MHC markers with respect to controls (Y-axis) are represented for Control **(A)**, C19wSepsis **(B)**, C19NoSepsis **(C)** and Sepsis Alone **(D)** cohorts. All data expressed as mean±SEM and statistical significance shown for data where *p* < 0.05. Note: Statistical significance level is indicated by the number of asterisks. ** *p* ≤ 0.01; *** *p* ≤ 0.001; ns, non-significant, p > 0.05.

The average values of the four cell death markers (caspase-1, caspase-3, MLKL and p62) two cytokines (IL-1-beta and IFN-gamma) and two MHCs (MHC I-A and MHC II-DRB1) of C19wSepsis, C19NoSepsis and Sepsis alone differed markedly from those of controls (**Figures 4**–**6**; **Table 2**).

ELISA results from control serum samples (from normal healthy subjects) showed no significant differences between the secreted levels of cell death markers (caspase-1, caspase-3, MLKL, and p62) in the control cohort (*p*>0.05), indicating comparable basal expression of the cell death markers (**Figure 4A**). The actual levels for the above intracellular cell death markers are shown in **Supplementary Table S1**. The mean fold-changes in the secreted levels of cell death markers in the control cohorts are summarized in **Table 2**.

C19wSepsis cohort showed noticeable differences among secreted cell death markers (**Figure 4B**; **Table 2**). A significant increase in the mean fold-change in the secreted levels of caspase-3 compared to that of caspase-1 (> 4-fold increase, ****p<0.0001), to that of MLKL (> 2-fold increase, ***p=0.0008), and to that of p62 (> 3-fold increase, ***p=0.0002) were observed in C19wSepsis cohort. While the secreted MLKL was not significantly different from that of p62 (*p*>0.05), the secreted caspase-1 in C19wSepsis cohort was lower than that of MLKL and p62 (*p*>0.05).

C19NoSepsis cohort demonstrated marked variability in the secreted levels of cell death markers (**Figure 4C**). A significant increase in the mean fold-change in the secreted levels of caspase-3 compared to that of caspase-1 (> 10-fold increase, ****p<0.0001), MLKL (> 2-fold increase, **p=0.0187), and p62 (> 13-fold increase, ****p<0.0001) was observed. While secreted caspase-1 was not significantly different from that of p62 (p>0.05), secreted MLKL was significantly higher than caspase-1 (> 4-fold increase, **p=0.0051) and p62 (> 6-fold increase, **p=0.0072).

Sepsis Alone cohort displayed distinct pattern among the secreted levels of cell death markers (**Figure 4D**). A significant increase in the mean fold-change in the secreted levels of caspase-3 compared to that of caspase-1 (> 7-fold increase, ****p<0.0001) and p62 (> 8-fold increase, ****p<0.0001). However, the secreted levels of caspase-3 were not significantly different from that of MLKL (*p*>0.05). While the secreted caspase-1 was not significantly different from that of p62 (p>0.05) in Sepsis Alone cohort, secreted MLKL was significantly higher than that of caspase-1 (> 6-fold increase, ***p=0.0004) and p62 (> 7-fold increase, ***p=0.0002).

The three clinical cohorts and control cohort showed distinct patterns in the secreted cytokines, IL-1-beta and IFN-gamma. There was no significant difference in the mean fold-change in the secreted levels of IL-1-beta and IFN-gamma (*p*>0.05), in the control cohort (**Figure 5A**). The secreted IL-1-beta was significantly higher than that of IFN-gamma (1.7-fold increase, **p=0.0046) in C19wSepsis cohort (**Figure 5B**). Observation of the cytokine data in the C19NoSepsis cohort also showed the secreted levels of IL-1-beta were significantly higher than that of IFN-gamma (> 2-fold increase, **p=0.0041) as shown in **Figure 5C**. The Sepsis Alone cohort showed significantly higher IFN-gamma levels compared to IL-1-beta (1.3-fold increase, **p=0.0041), as shown in **Figure 5D**.

The three clinical conditions and control cohort showed noticeable variability in the secreted MHC I-A and MHC II-DRB1. Secreted levels of MHCs in the control cohort showed a significant difference in MHC I-A versus MHC II-DRB1 levels, with the latter showing higher levels in control subjects (***p=0.0002) (**Figure 6A**). A comparison of these two MHCs within the C19wSepsis cohort showed that the mean fold-change in the secreted levels of MHC I-A was significantly higher than that of MHC II-DRB1 (1.7-fold increase, **p=0.0015) (**Figure 6B**). The secreted MHC I-A levels in C19NoSepsis and Sepsis Alone cohorts were comparable to MHC II-DRB1, with no significant difference observed between the two MHCs in these two cohorts (*p*>0.05) (**Figures 6C, D**).

### 3.3 Intracellular cell death markers between clinical cohorts

**Figure 7:**
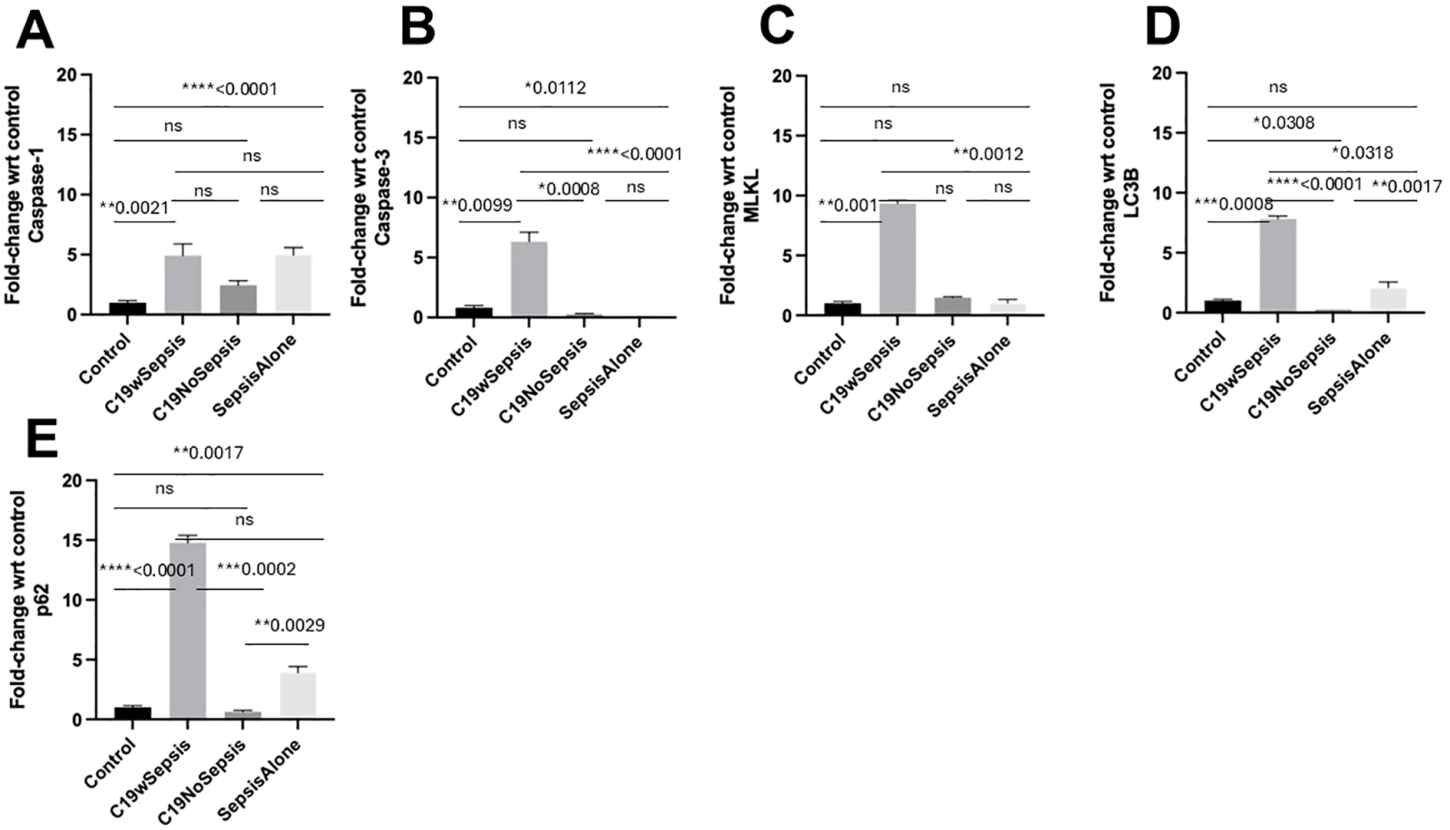
Flow cytometry analysis of intracellular cell death markers between clinical cohorts. Normalized flow cytometry data of PBMC samples from Control, C19wSepsis, C19NoSepsis and Sepsis Alone cohorts. Clinical cohorts (X-axis) versus fold-change in intracellular levels of cell death markers with respect to controls (Y-axis) are represented for caspase-1 **(A)**, caspase-3 **(B)**, MLKL **(C)**, LC3B **(D)** and p62 **(E)**. All data expressed as mean±SEM and statistical significance shown for data where *p* < 0.05. Note: Statistical significance level is indicated by the number of asterisks. * *p* ≤ 0.05; ** *p* ≤ 0.01; *** *p* ≤ 0.001; **** *p* ≤ 0.0001; ns, non-significant, *p* > 0.05.

The mean fold-change in % gated cell population with respect to controls for the four cell death markers (caspase-1, caspase-3, MLKL and p62), two cytokines (IL-1-beta and IFN-gamma) and two MHCs (MHC I-A and MHC II-DRB1) observed for C19wSepsis, C19NoSepsis and Sepsis Alone clinical cohorts differed markedly in intracellular molecular signatures from those observed for the control cohort (**Figure 7**; **Table 3**).

The intracellular levels of caspase-1 varied among different cohorts and controls (**Figure 7A**; **Table 3**). While a significant increase in the mean fold-change in intracellular caspase-1 levels in the C19wSepsis (> 4-fold increase, **p=0.0021) and Sepsis Alone cohorts (> 4-fold increase, ****p<0.0001) were observed compared to healthy controls, C19NoSepsis cohort showed no significant change (*p*>0.05). No significant differences in the intracellular caspase-1 between the clinical conditions of C19wSepsis, C19NoSepsis, and Sepsis Alone (*p*>0.05) were observed.

The intracellular caspase-3 levels showed prominent differences among different cohorts and controls. A significant increase in the mean fold-change in intracellular caspase-3 levels in C19wSepsis patients compared to healthy controls (> 7-fold increase in C19wSepsis, **p=0.0099) was observed, while a significant decrease was noted in the Sepsis Alone cohort (> 5-fold decrease in Sepsis Alone, *p=0.0112) (**Figure 7B**; **Table 3**). C19NoSepsis cohort showed no significant decrease (*p*>0.05). Comparison of the intracellular levels of caspase-3 between the clinical conditions showed a significant increase in the intracellular caspase-3 in the C19wSepsis cohort compared to the C19NoSepsis (> 22-fold increase, ***p=0.0008) and Sepsis Alone (> 39-fold increase, ****p<0.0001) cohorts. However, there was no significant difference in the intracellular caspase-3 between the C19NoSepsis and Sepsis Alone cohorts (*p*>0.05).

The intracellular MLKL levels varied markedly among different cohorts and controls. A significant increase in the mean fold-change in intracellular levels of MLKL in the C19wSepsis cohort compared to that in healthy controls was observed (> 9-fold increase, **p=0.001), while the other two cohorts, namely the C19NoSepsis and Sepsis Alone cohorts, showed comparable and no significant difference in the intracellular levels of MLKL when compared to that in healthy controls (*p*>0.05) (**Figure 7C**; **Table 3**). Comparison of the intracellular MLKL levels between the clinical conditions showed a significant increase in the intracellular expression of MLKL in the C19wSepsis cohort compared to Sepsis Alone (> 6-fold increase, **p=0.0012), but not when compared to the C19NoSepsis cohort (p>0.05). There was no significant difference in the intracellular MLKL between the C19NoSepsis and Sepsis Alone cohorts (p>0.05).

The intracellular LC3B levels showed a noticeable pattern among different cohorts and controls. The mean fold-change in intracellular LC3B levels in the C19wSepsis cohort were significantly higher compared to that in healthy controls (> 7-fold increase, ***p=0.0008); in contrast, the intracellular LC3B levels in the C19NoSepsis cohort showed a significant decrease (> 3-fold decrease, *p=0.0308), while the Sepsis Alone cohort showed an increase in intracellular LC3B but no significant difference when compared to that in healthy controls (*p*>0.05) (**Figure 7D**; **Table 3**). A comparison of the intracellular LC3B levels between the clinical conditions showed a significant increase in the C19wSepsis cohort compared to that in the C19NoSepsis (> 30-fold increase, ****p<0.0001) and Sepsis Alone (> 3-fold increase, *p=0.0318) cohorts. There was a significant increase in the intracellular LC3B in the Sepsis Alone cohort compared to the C19NoSepsis cohort (> 8-fold increase, **p=0.0017).

The intracellular p62 levels demonstrated a distinct pattern among different cohorts and controls. The mean fold-change in intracellular p62 levels were significantly elevated when compared to the healthy controls in the C19wSepsis cohort (> 14-fold increase, ****p<0.0001) and Sepsis Alone cohort (> 3-fold increase, **p=0.0017), while the C19NoSepsis cohort showed no significant difference in the mean fold-change in the intracellular levels of p62 when compared to healthy controls (*p*>0.05) (**Figure 7E**; **Table 3**). Comparison of the intracellular p62 levels between the clinical conditions showed a significant increase in the C19wSepsis cohort compared to that in the C19NoSepsis cohort (> 22-fold increase, ***p=0.0002) but not in the Sepsis Alone cohort (*p*>0.05). There was a significant increase in the intracellular levels of p62 in the Sepsis Alone cohort compared to that in the C19NoSepsis cohort (approximately a 6-fold increase, **p=0.0029).

### 3.4 Intracellular cell death markers within each clinical cohort

**Figure 8:**
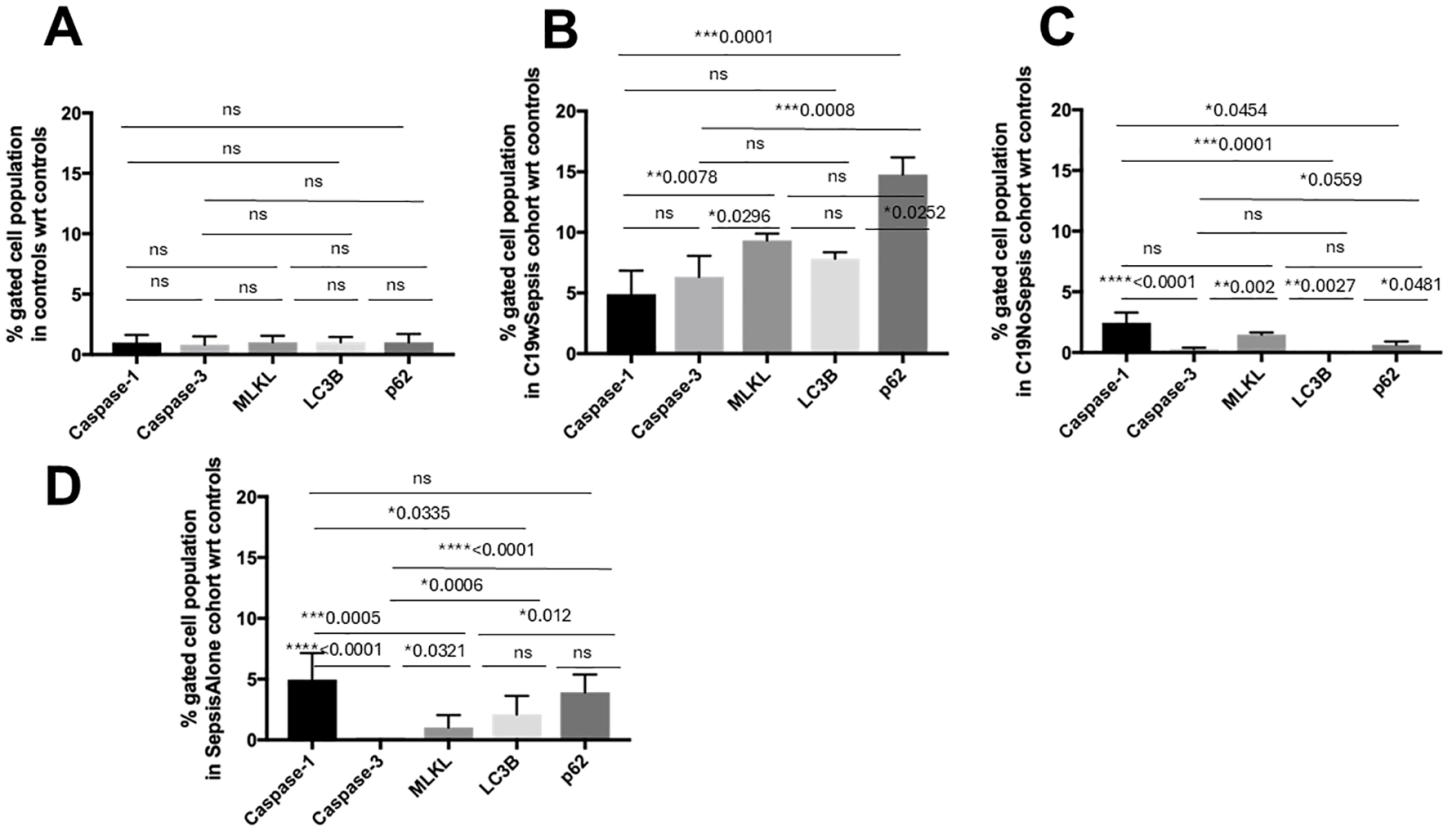
Flow cytometry analysis of intracellular cell death markers within each clinical cohort. Normalized flow cytometry data of PBMC samples from Control, C19wSepsis, C19NoSepsis and Sepsis Alone cohorts. Cell death markers (X-axis) versus fold-change in intracellular levels of cell death markers with respect to controls (Y-axis) are represented for Control **(A)**, C19wSepsis **(B)**, C19NoSepsis **(C)** and Sepsis Alone **(D)** cohorts. All data expressed as mean±SEM and statistical significance shown for data where *p* < 0.05. Note: Statistical significance level is indicated by the number of asterisks. * *p* ≤ 0.05; ** *p* ≤ 0.01; *** *p* ≤ 0.001; **** *p* ≤ 0.0001; ns, non-significant, *p* > 0.05.

**Figure 9:**
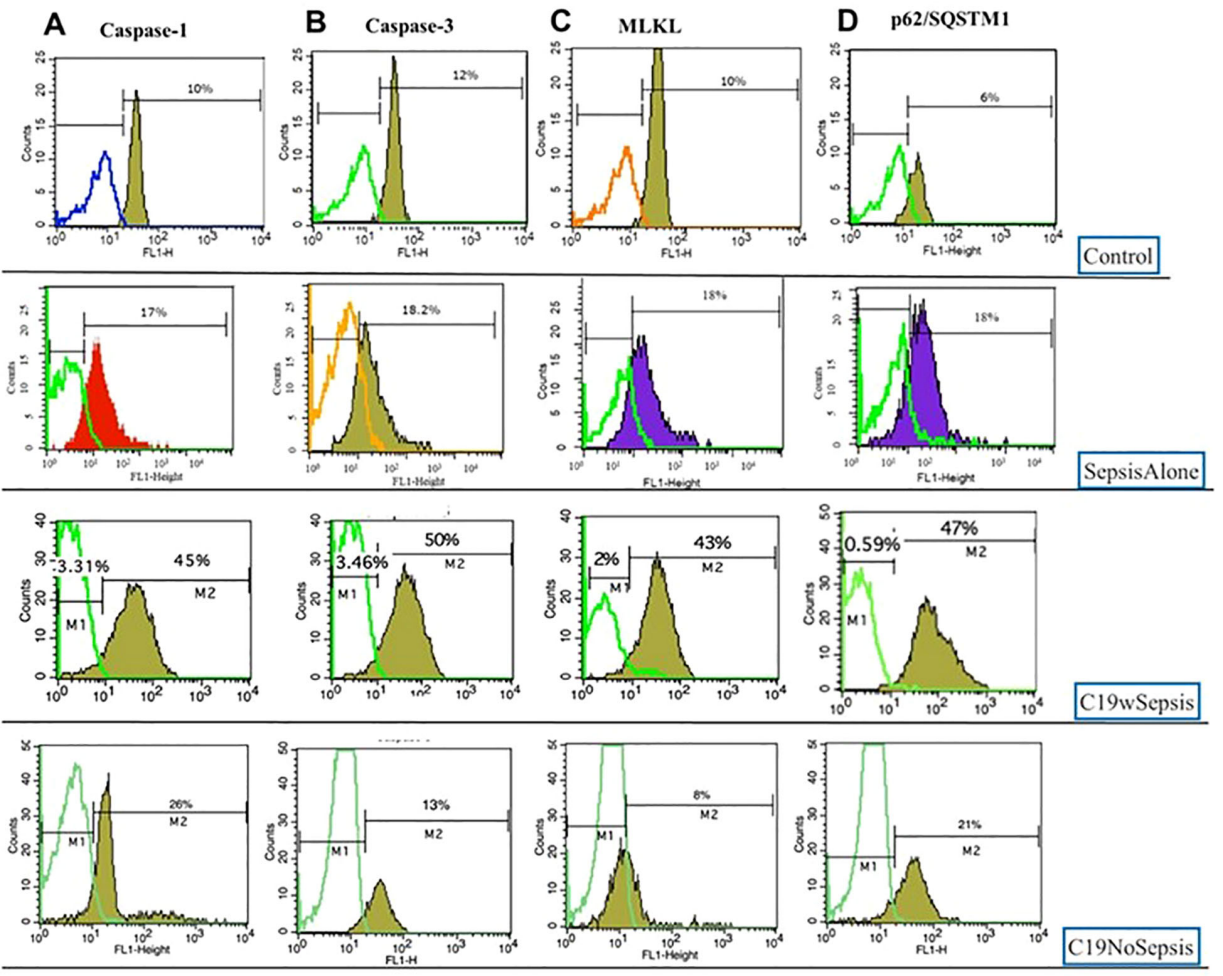
Representative histogram plots of Caspase-1 **(A)**, Caspase-3 **(B)**, MLKL **(C)** and p62 **(D)** derived from flow cytometry analysis of PBMCs derived from Control, Sepsis Alone, C19wSepsis and C19NoSepsis cohorts.

The control cohort showed comparable intracellular basal expression levels of caspase-1, caspase-3, MLKL, LC3B, and p62 in the healthy controls as shown in **Figure 8A**. The mean fold-changes in the levels of caspase-1, caspase-3, MLKL, p62, and LC3B were not significantly different (*p*>0.05) in the control subjects. Actual levels for the above intracellular cell death markers are shown in **Supplementary Table S2**. Histogram plots and data for the mean fold-change in the intracellular levels of cell death markers in control subjects are shown in **Figure 9**; **Table 3**, respectively.

C19wSepsis cohort markedly varied in the intracellular expression of cell death markers (**Figure 8B**; **Table 3**). The mean fold-change data with respect to controls showed significantly lower intracellular caspase-1 levels compared to those of MLKL (> 1.9-fold increase in MLKL, **p=0.0078) and p62 (> 3-fold increase in p62, ***p=0.0001) (**Figure 8B**; **Table 3**). However, intracellular caspase-1 was not significantly different from caspase-3 and LC3B (*p*>0.05). Intracellular caspase-3 levels were significantly lower than those of MLKL (> 1.5-fold increase in MLKL, *p=0.0296) and p62 (> 2-fold increase in p62, ***p=0.0008). However, intracellular caspase-3 was not significantly different from LC3B (*p*>0.05) and intracellular MLKL was not significantly different from LC3B and p62 (*p*>0.05). Intracellular LC3B levels were significantly lower than p62 (> 1.9-fold increase in p62, *p=0.0252).

C19NoSepsis cohort showed noticeable differences among intracellular cell death markers (**Figure 8C**; **Table 3**). The mean fold-change data with respect to controls showed significantly higher intracellular caspase-1 levels compared to those of caspase-3 (> 8-fold increase in caspase-1, ****p<0.0001), LC3B (> 9-fold increase in caspase-1, ***p=0.0001), and p62 (> 3-fold increase in caspase-1, *p=0.0454). However, the intracellular caspase-1 levels were not significantly different from MLKL levels (*p*>0.05). Intracellular MLKL level was significantly different from caspase-3 (> 5-fold increase in MLKL, **p=0.002) and LC3B (> 5-fold increase in MLKL, **p=0.0027), and not significantly different from p62 (*p*>0.05). The intracellular LC3B levels were significantly different from those of p62 (> 2-fold increase in p62, *p=0.0481). No significant differences were observed between intracellular caspase-3, LC3B, and p62 (*p*>0.05).

Sepsis Alone cohort displayed distinct differences among intracellular cell death markers (**Figure 8D**; **Table 3**). The mean fold-change data with respect to controls showed significantly higher intracellular caspase-1 levels compared to that of caspase-3 (> 30-fold increase in caspase-1, ****p<0.0001), MLKL (> 3-fold increase in caspase-1, ***p=0.0005), and LC3B (> 2-fold increase in caspase-1, *p=0.0335) but not p62. Intracellular caspase-3 levels were significantly different from MLKL (> 8-fold increase in MLKL, *p=0.0321), LC3B (> 13-fold increase in LC3B, ***p=0.0006), and p62 (> 20-fold increase in p62, ****p<0.0001). Also, intracellular MLKL was significantly different from p62 (> 2-fold increase in p62, *p=0.012). However, no significant difference was found between intracellular levels of MLKL and LC3B or between LC3B and p62 (*p*>0.05).

## 4 Discussion

The current study investigated cell death markers, cytokines and MHCs in COVID-19 with and without sepsis (C19wSepsis and C19NoSepsis) and Sepsis Alone cohorts compared to healthy controls. Secreted and intracellular levels of caspase-1, caspase-3, MLKL, LC3B, and p62 in healthy controls were comparable, indicating no difference in the basal expression of cell death markers. Similarly, the secreted levels of IL-1-beta and IFN-gamma in the control subjects were comparable in the current study.

Intracellular caspase-3 levels were significantly higher in the C19wSepsis cohort than in the control, C19NoSepsis, and Sepsis Alone cohorts (**Figure 8**; **Table 3**). Additionally, the C19wSepsis, C19NoSepsis and Sepsis Alone cohorts showed a significant increase in the secreted levels of caspase-3 compared to controls. Single-cell RNA-seq analysis showed elevated caspase-3 levels in red blood cells isolated from COVID-19 patients compared with controls (42). However, another study showed patients with COVID-19 infection had significantly higher levels of caspase-3 in serum than controls (43). The general trend among the cell death markers in the current study, as reflected in the three patient cohorts (C19wSepsis, C19NoSepsis and Sepsis Alone) is that secreted levels of caspase-3 were significantly higher than one or more of the non-apoptotic cell death markers. Conversely, intracellular levels of caspase-3 were lower than those of one or more non-apoptotic cell death markers. Elevated serum caspase-3 levels are associated with sepsis severity and mortality (27). This may indicate that COVID-19 susceptible individuals (who were also survivors in this study) may have had apoptotic immune cell death compensated by non-apoptotic cell death mechanisms during the protective anti-microbial immune response to pathogen invasion.

Intracellular caspase-1 significantly increased in the C19wSepsis and Sepsis Alone cohorts, with no significant increase in the C19NoSepsis cohort compared to controls (**Figure 8**; **Table 3**). The intracellular caspase-1 levels were higher in the sepsis cohort than the control cohort as shown in our earlier studies (23). The secreted levels of caspase-1 were significantly lower in the C19NoSepsis cohort than in the other cohorts. It is possible that caspase-1 is secreted into microvesicles as reported earlier, and caspase-1 was shown to regulate lymphocyte apoptosis in sepsis (24). Single-cell RNA-seq analysis showed upregulation of caspase-1 in CD4+ T cells from COVID-19 hospitalized patients compared to unexposed controls (42). Additionally, patients with long-term COVID-19 symptoms also showed upregulated caspase-1 activity in CD4+ T-cells (42). Exosomes isolated from hospitalized, severe COVID-19 patients, when exposed to human microvascular endothelial cells, significantly stimulated the mRNA expression of the inflammasome, caspase-1, and the pleiotropic cytokine, IL-1-beta. Exosomes from mild COVID-19 patients and healthy controls did not show significant changes in the expression of the same markers (44). This indicates that disease severity and clinical heterogeneity play key roles in the immune regulatory functions of cell death markers in COVID-19 infections. However, a prospective longitudinal study that investigated inflammasome activation in patients with COVID-19 and bacterial septic shock in ICU showed a decrease in caspase-1 levels in neutrophils compared to healthy controls, indicating a cell-specific regulatory role of caspase-1-mediated pyroptosis (45).

Intracellular MLKL levels showed a significant increase in the C19wSepsis and C19NoSepsis cohorts compared to the healthy control and Sepsis Alone cohorts (**Figure 8**; **Table 3**). Intracellular MLKL levels were significantly higher in the sepsis cohort than in the control cohorts as shown in our previous study (23). Additionally, a significant increase in the secreted levels of MLKL was observed in the Sepsis Alone cohort compared to the control group. Serum samples from severely infected COVID-19 patients that experienced major cardiovascular adverse events showed elevated levels of the active form of MLKL, phospho-MLKL (46). Additionally, a major increase in the expression of MLKL-related genes and genes implicated in necroptotic cell death in severe type compared to mild type COVID-19 patients was observed, highlighting the significant role necroptotic cell death plays in disease severity (46).

The intracellular p62 levels in the C19wSepsis and Sepsis Alone cohorts were significantly higher than those in the healthy controls and C19NoSepsis cohorts (**Figure 8**; **Table 3**). A significant increase in intracellular levels of p62 was observed in the sepsis cohort compared to that in healthy controls as shown in our previous study (23). A significant decrease in the secreted levels of p62 levels was observed in the C19NoSepsis and Sepsis Alone cohorts compared to the controls. There was a significant decrease in secreted levels of p62 in the sepsis group compared to healthy controls as shown in our previous study (23). Additionally, the C19wSepsis cohort showed significantly higher levels of intracellular LC3B than the C19NoSepsis and Sepsis Alone cohorts did. While the downregulation of intracellular p62 by SARS-CoV-2 might promote viral replication by preventing antiviral immunity in the host, an increase in p62 levels might be anti-inflammatory triggering autophagy-dependent blockade of the infection process (47). This indicates that autophagy mechanisms either have a pro- or anti-inflammatory role depending on the immunological status of the patient and the duration of infection. The significant increase in p62 levels in C19wSepsis and Sepsis Alone survivors in the current study may reflect the robust immunoprotected mechanisms observed in these immunocompetent patient cohorts. The current study highlights the effect of PCD mechanisms on the disease severity and clinical outcomes of COVID-19 infections. The limitations of the current study include sample size, paucity of sample volume, and clinical heterogeneity including comorbidities, vaccination status, infection with different variants of SARS-CoV-2 between the patient cohorts.

A significant increase in IFN-gamma levels in the C19wSepsis and Sepsis Alone cohorts was observed compared to the control and C19NoSepsis cohorts in the current study. Secreted levels of IL-1-beta were significantly higher than IFN-gamma in the C19wSepsis and C19NoSepsis cohorts, indicative of caspase-1-mediated activation of IL-1-beta during the antiviral immune response mechanism (**Figure 5**; **Table 2**). The characteristic hyperinflammatory immune response during COVID-19-induced sepsis is the systemic cytokine storm syndrome triggered in the aftermath of exacerbated antiviral immunity, which contributes to inflammatory cell death (31). There is an interplay between the classical (apoptotic) and non-classical (necroptotic, pyroptotic, and autophagic) forms of PCD; for example, both necroptosis and autophagy are known to modulate pyroptotic cell death (32, 33). Pyroptosis was reported to regulate the release of proinflammatory cytokines, IL-1-beta and IL-18 in COVID-19 and bacterial sepsis (45). Notably, it was also demonstrated that the activation of IL-1-beta was dependent on disease severity (48). Significantly higher levels of IL-1-beta and TNF-alpha were observed in the sera of patients with COVID-19 infection than in controls (43). However, literature reports also demonstrated that only a few of the COVID-19-infected patients to have displayed the characteristic cytokine storm syndrome; most of them had lower cytokine profiles, reduced interferon signaling, and markedly reduced circulating T cell counts, indicative of an impaired effector immune response (38, 39). Preferential mechanisms of cytokine responses were observed in patients with severe versus moderate COVID-19 patients, with IFN-alpha-mediated responses being more prevalent in the former subset, while TNF-alpha plays a role in the latter (49).

The C19wSepsis cohort showed significantly higher levels of MHC I-A than MHC II-DRB1 in the current study (**Figure 6**; **Table 2**). The marked elevation of MHC levels seen in the current study, especially in the C19wSepsis cohort compared to the C19NoSepsis, Sepsis Alone, and control cohorts, emphasizes the protective role of MHCs in boosting the host antiviral immune response against SARS-CoV-2 infection in these patient survivors of the pandemic. Comparison of the two classes of MHCs within the C19wSepsis cohort showed significantly higher levels of MHC I-A than over MHC II-DRB1, further underlining the significance of MHC Class I family members in the protective immune response to viral infection-induced sepsis in moderately infected patient survivors. The association of MHC I and MHC II expression (or their gene expression) is negatively correlated with SARS-CoV-2 infection, and hindrance to MHC expression evades T cell recognition (39, 50, 51). The variability in MHC levels in SARS-CoV-2 disease pathogenesis and severity, with and without sepsis, requires further investigation.

Findings from the current study are useful in designing studies with larger sample sizes that would help stratify patients based on factors contributing to clinical heterogeneity, the severity of sepsis and survivors versus non-survivors of sepsis. Further studies are needed to understand the effect of the co-existence of PCD mechanisms on sepsis with underlying comorbidities, which would also help define the clinical utility of PCD biomarkers for early diagnosis and appropriate therapeutic interventions.

## 5 Conclusions

This study identifies two distinct molecular signatures associated with disease severity in COVID-19 patients, with and without sepsis. The first signature, characterized by the differential expression of secreted markers, was present in both the C19wSepsis and C19NoSepsis cohorts. The second signature, indicated by the expression of intracellular PCD markers (including caspase-1, caspase-3, MLKL, p62/SQSTM1), was exclusive to the C19wSepsis cohort. These findings suggest that the cellular marker signature is specifically linked to sepsis in COVID-19 patients, providing a potential diagnostic tool to differentiate sepsis-related immune responses from other inflammatory states. Understanding these molecular signatures may aid in the development of targeted therapeutic strategies and improve the management of COVID-19 patients in critical care settings.

## Data Availability

The original contributions presented in the study are included in the article/**Supplementary Material**. Further inquiries can be directed to the corresponding authors.
