# Supplementary material for: Programmed cell death markers in COVID-19 survivors with and without sepsis
Source: Front Immunol. 2025 Feb 20;16:1535938. doi: 10.3389/fimmu.2025.1535938 (PMC11882558; doi:10.3389/fimmu.2025.1535938)
Supplement: Supplementary file 1 [file Table1.docx]

Supplementary Data Table #1: Normalized ELISA data from serum samples of control cohort.

|  |  | Caspase-1 | Caspase-3 | MLKL | p62 |
| --- | --- | --- | --- | --- | --- |
|  | N | 18 | 15 | 21 | 18 |
|  | Mean | 1 | 1 | 1 | 1 |
|  | Std. Error of Mean | 0.1533 | 0.16 | 0.1054 | 0.1426 |
|  |  |  |  |  |  |
|  |  |  |  |  |  |
|  |  | Caspase-1 | Caspase-3 | MLKL | p62 |
|  | 1 | 0.105006 | 0.201625 | 1.090102 | 0.987758 |
|  | 2 | 1.234232 | 0.186814 | 1.288747 | 1.105964 |
|  | 3 | 0.645696 | 0.127802 | 0.947646 | 0.844411 |
|  | 4 | 1.07418 | 0.5951 | 0.633388 | 0.956822 |
|  | 5 | 0.732254 | 0.5951 | 1.296475 | 1.33364 |
|  | 6 | 1.480805 | 1.582082 | 1.067458 | 1.207534 |
|  | 7 | 0.15385 | 1.237856 | 0.647761 | 0.026921 |
|  | 8 | 0.246663 | 1.082292 | 0.962535 | 0.115094 |
|  | 9 | 1.563953 | 1.754377 | 0.077094 | 1.33364 |
|  | 10 | 1.011888 | 1.221365 | 0.583269 | 1.099831 |
|  | 11 | 2.0306 | 1.163862 | 0.298799 | 1.840156 |
|  | 12 | 1.317522 | 0.977255 | 1.078773 | 1.191225 |
|  | 13 | 1.685528 | 1.38752 | 1.269457 | 2.305012 |
|  | 14 | 1.234232 | 2.280255 | 1.591044 | 1.398668 |
|  | 15 | 1.974564 | 0.606695 | 1.277168 | 1.137002 |
|  | 16 | 1.288436 |  | 1.440619 | 0.870388 |
|  | 17 | 0.121887 |  | 1.146959 | 0.112266 |
|  | 18 | 0.098704 |  | 1.59903 | 0.133667 |
|  | 19 |  |  | 1.915893 |  |
|  | 20 |  |  | 0.243693 |  |
|  | 21 |  |  | 0.544089 |  |
|  |  |  |  |  |  |
