# Supplementary material for: Programmed cell death markers in COVID-19 survivors with and without sepsis
Source: Front Immunol. 2025 Feb 20;16:1535938. doi: 10.3389/fimmu.2025.1535938 (PMC11882558; doi:10.3389/fimmu.2025.1535938)
Supplement: Supplementary file 2 [file Table2.docx]

Supplementary Data Table #2: Normalized flow cytometry data from PBMC samples of control cohort.

|  | Caspase-1 | Caspase-3 | MLKL | LC3B | p62 |
| --- | --- | --- | --- | --- | --- |
| N | 14 | 16 | 14 | 15 | 18 |
| Mean | 1 | 0.8125 | 1 | 1 | 1 |
| Std. Error of Mean | 0.1685 | 0.1759 | 0.15 | 0.1176 | 0.1667 |
|  |  |  |  |  |  |
|  |  |  |  |  |  |
|  | Caspase-1 | Caspase-3 | MLKL | LC3B | p62 |
| 1 | 1.69692652 | 0.60494426 | 0.99347991 | 1.41736312 | 1.00645161 |
| 2 | 1.81375941 | 2.75060591 | 0.09552691 | 1.2050145 | 0.2568915 |
| 3 | 0.73376756 | 0.69316529 | 0.10614102 | 0.39655466 | 0.31319648 |
| 4 | 2.37370242 | 0.582889 | 0.61561789 | 1.08476889 | 0.57008798 |
| 5 | 0.56991655 | 0.09767329 | 1.00409401 | 0.68565581 | 0.91495601 |
| 6 | 0.30063098 | 0.8349491 | 0.62835481 | 1.33293536 | 0.3026393 |
| 7 | 0.84062691 | 0.81919535 | 1.06141016 | 0.70100631 | 1.36891496 |
| 8 | 0.86912274 | 0.88221037 | 1.10811221 | 0.19443971 | 1.44633431 |
| 9 | 1.01160187 | 0.81919535 | 1.23335861 | 0.79310933 | 0.06334311 |
| 10 | 0.71239568 | 1.74866699 | 1.1802881 | 0.51168344 | 2.15014663 |
| 11 | 1.43903928 | 1.13427048 | 2.37755876 | 1.30479277 | 1.12609971 |
| 12 | 0.78363525 | 1.0555017 | 1.40530705 | 1.84206038 | 1.40762463 |
| 13 | 0.00000001 | 0.97673291 | 1.169674 | 1.53505032 | 1.47800587 |
| 14 | 0.85487482 | 0.00000001 | 1.02107657 | 1.02336688 | 0.63343109 |
| 15 |  | 0.00000001 |  | 0.97219853 | 2.81524927 |
| 16 |  | 0.00000001 |  |  | 1.05571848 |
| 17 |  |  |  |  | 0.49266862 |
| 18 |  |  |  |  | 0.59824047 |
